# Investigating interaction pattern between urban-rural integration and transport network: A dynamic evolution model

**DOI:** 10.1371/journal.pone.0266063

**Published:** 2022-05-04

**Authors:** Daiquan Xiao, Xuecai Xu

**Affiliations:** School of Civil and Hydraulic Engineering, Huazhong University of Science and Technology, Wuhan, China; Tongji University, CHINA

## Abstract

With the progress of urbanization and urban-rural integration (URI), the interaction between URI and urban-rural transport network is becoming stronger, thus it is necessary to investigate the interactive relationship between URI and transport network. By assuming certain functions and principles, evolution model was proposed to explain the relationship between URI space development scale and urban-rural transport network scale, and then by considering the interaction between URI and transport network, dynamic evolution model was presented. Stability analysis was performed to verify the interaction relations with three patterns and elasticity simulation provided the optimal value of urban-rural transport network scale configuration. The results may provide potential insights on URI development and transport network.

## Introduction

With the implementation of “Transport Power” strategy in China and rapid developing phase of urbanization, urban-rural integration (URI) has become the main orientation, and more URI developing regions and areas will be formed surrounding metropolitan cities. Throughout the worldwide, metropolitan areas, e.g. Tokyo, New York, have experienced the URI developing phase and then formed into megapopolis, while a lot of cities in China are experiencing the URI period now.

Conceptually, URI represents that driven by the urban development, each social undertakings and urban development form interactive pattern within certain areas of surrounding regions, which breaks the urban-rural dual structure, and mixes together so as to form into the pattern of urban promoting rural development whilst rural supporting urban development. On the other side, urban-rural transport network is defined as the general transport network within urban and rural regions governed whereas in the narrow sense it is denoted as the transport channel of urban-rural areas.

In the developing history of world cities, there exists a close relationship between city space expansion and transport network, both of which influence and restrain each other. Transport network provides necessary supporting fundamentals for URI, and leads city space expansion and industry layout, while URI promotes the developing progress of transport network, and provides economic support and demand orientation, thus there exists interactive and inter-feedback relationship between URI and transport network. However, shown from the progress of urbanization and URI, URI construction and transport network developing can’t match with each other exactly since both have its own developing and evolution principle. Therefore, according to the evolvement theory, through interactive and steadily ordered system intrinsically, how to construct an interactive model between URI and transport network is critical to solve the relevant transportation issues during URI process.

## Literature review

Over the last two decades, different studies about urbanization, urban-rural integration and transport network separately have been investigated, and the literature review concentrates on work related to three aspects above.

How far has urbanization gone? Various studies have verified this. Peng et al. (2016) [1] presented a Relative Rurality Index (RRI) to characterize the integrated transformation during China’s process of urbanization with an emphasis on urban-rural integration. The non-linear regression analysis showed that urban-rural system had undergone deep and lasting change, and the rurality by RRI was more accurate and comprehensive than the urbanization level. Zeng et al. (2019) [2] explored the spatial spillover influence of infrastructure network on urbanization. It was found that rational utilization of the embedded spatial spillover effect can help to formulate strategies for sustainable urbanization. Identical study by Ji et al. (2019) [3] employed a hybrid heterogeneous data envelopment analysis method to guide sustainable urbanization completion. Based on the quality and scale of urbanization, Shi et al. (2020) [4] constructed an evaluation index system for urbanization coordination level. By introducing the spatial econometric regression model, the results showed that the spatial and temporal patter of urbanization was affected by internal source, administrative level and investment level. Similarly, Ge et al. (2021) [5] evaluated the spatiotemporal distribution characteristics of urbanization levels and traffic accessibility with bivariate spatial autocorrelation model and spatial regression models. It was found that there existed a positive relation between urbanization level and traffic accessibility, but with a significant scale effect. The recent study by Chen et al. (2021) [6] summarized the classic theories and studies on urban-rural relations and proposed some issues regarding integration of new-type urbanization and rural revitalization from different perspectives, which provided some trends for high-quality development of urban and rural areas in the new era.

URI plays an important role in urbanization process. Li (2012) [7] proposed specific ways of achieving URI corresponding to different urban-rural interaction patterns. An urban-rural interaction index was put forward to categorize into three groups, which was dealt with from the perspectives of dynamic land use. Zhao (2013) [8] stated that URI strategy played a positive role in improving living conditions in rural areas and reducing the social and economic gaps, but it would not be easy the achieve the goal of urban-rural integration and harmony society. Yan et al. (2018) [9] established a theoretical framework to analyze the formation of urban-rural mixed community by considering the entire cell as a grid unit, and the results revealed that urban-rural mixed community can be regarded as a micro-unit in achieving URI. Recent study by Yang et al. (2021) [10] developed a conceptual framework based on the URI system, and URI index was proposed to measure the regional differentiation. It was found that the overall URI in China kept a low level, and spatial agglomeration from URI index showed as high in the east and low of the western and central regions.

URI is not only highly concerned with urbanization, but also with other factors, e.g. land condition, industry activity, and transportation situation. Wu and Cui (2016) [11] explored the time-space evolution characteristics of URI development with GIS technology, and the results indicated that URI revealed a spatial imbalance and transportation situation affected URI development. Similar studies by Li et al. (2017) [12] and Du et al. (2021) [13] investigated the spatial-temporal characteristics and driving mechanism of rural transformation development. He et al. (2019) [14] proposed the spatial organization pattern of URI in urban agglomerations in China, and an urban-rural multi-level polycentric network was built to guide the optimization of URI with an emphasis on the three-level centers of city-town-village and networking linkages.

To sum up, most of the studies are concentrated on urbanization and URI, and a few are focused on the relationship between URI and transportation, whereas there is lack of how to establish the relationship between the URI scale and transport network. Therefore, the purpose of this study is to explore the interaction between URI scale and transport network, and determine the optimal URI scale and transport network so that the results may provide potential insights on travel demand and network optimization for urban-rural regions.

## Evolution model of URI and transport network

During the process of URI, the range of URI development space is larger than the urban area, and the evolution process is similar with transport accessibility since more transport network represents higher accessibility. Thus, the development levels of URI and transport network conform to the growing principles, which can be reflected by the growth functions.

URI development is meant to expand the space to the non-urban areas, and its accessibility and transport network scale match with URI development level, which can be described by Logistics model. On one hand, the URI scale and transport network in the evolution process are affected by non-technical factors, such as humanitarian, society, national conditions, policies, etc.; on the other hand, URI scale and transport network are influenced by themselves, and varied by phase and period. Therefore, independent evolution model for URI scale and transport network can be constructed so as to analyze the periodical evolution relations under certain conditions.

### A. Assumptions

1. The completion degree of transport network is represented by urban-rural accessibility generally while network density can intuitively characterize accessibility level; URI level is represented by URI space development scale while the scale is expressed by radius or area of URI region.
2. The completion degree of transport network is proportional to urban-rural accessibility, i.e. the higher the accessibility is, the higher the completion degree of transport network is, and the larger the scale of transport network is; Similarly, the URI space development scale is proportional to the development level, whose varying trend is close to each other.
3. The scales of URI space development and urban-rural transport network are associated with time, and both can be expressed as differential functions;
4. The levels of URI scale and urban-rural transport network follow the Logistic evolution principles.

### B. Evolution model

In accordance with the analysis and assumptions, the models of URI space development scale and urban-rural transport network scale can be expressed as:

$$\left\{\begin{array}{c} \frac{dy_{1}}{dt}=\alpha_{1}\beta_{1}\theta_{1}\left(1-\frac{y_{1}}{s_{1}}\right)y_{1}=k_{1}y_{1}\left(1-\frac{y_{1}}{s_{1}}\right)\\ \frac{dy_{2}}{dt}=\alpha_{2}\beta_{2}\theta_{2}\left(1-\frac{y_{2}}{s_{2}}\right)y_{2}=k_{2}y_{2}\left(1-\frac{y_{2}}{s_{2}}\right) \end{array}\right.$$ (1)

where *y*_1_ and *y*_2_ represent the actual scales of urban-rural transport network and URI space at time *t*, respectively; *s*_1_ and *s*_2_ denote the maximal scales of urban-rural transport network and URI scale without being influenced by each other, respectively; *α*_1_ and *α*_2_ stand for the original growth rates of URI development and transport routes in the process of transport network, respectively; *β*_1_ and *β*_2_ express the impact coefficient of previous transport network level on successive transport network development, and impact coefficient of previous URI scale on successive URI scale, respectively, in which $\beta_{1}=\frac{s_{1}-y_{1}^{0}}{s_{1}}$, and $\beta_{2}=\frac{s_{2}-y_{2}^{0}}{s_{2}}$, $y_{1}^{0}$ and $y_{2}^{0}$ denote transport network and URI scale at the initial stage; *θ*_1_ and *θ*_2_ are impact coefficients of external factors (including economics, humanitarian, natural and policies) on urban-rural transport network development and URI scale, respectively; *k*_1_ is dynamics factor of urban-rural transport network, *k*_1_ = *α*_1_*β*_1_*θ*_1_; *k*_2_ is dynamics factor of URI scale, *k*_2_ = *α*_2_*β*_2_*θ*_2_.

By solving the equations above, y_1_ and y_2_ can be obtained as followings:

$$\left\{\begin{array}{c} y_{1}=\frac{s_{1}}{1+\rho_{1}e^{-k_{1}t}}\\ y_{2}=\frac{s_{2}}{1+\rho_{2}e^{-k_{2}t}} \end{array}\right.$$ (2)

where $\rho_{1}=\frac{s_{1}y_{1}^{0}}{s_{1}-y_{1}^{0}}$, and $\rho_{2}=\frac{s_{2}y_{2}^{0}}{s_{2}-y_{2}^{0}}$

### C. Dynamic evolution model

Based on the previous Eq (1), and combined the Logistic evolution principle, the dynamic models between URI and urban-rural transport network can be expressed as non-linear differential equations:

$$\left\{\begin{array}{c} \frac{dy_{1}}{dt}=\alpha_{1}\beta_{1}\theta_{1}\left(1-\frac{y_{1}}{s_{1}}+\gamma_{1}\frac{y_{2}}{s_{2}}\right)y_{1}=k_{1}y_{1}\left(1-\frac{y_{1}}{s_{1}}+\gamma_{1}\frac{y_{2}}{s_{2}}\right)\\ \frac{dy_{2}}{dt}=\alpha_{2}\beta_{2}\theta_{2}\left(1-\frac{y_{2}}{s_{2}}+\gamma_{2}\frac{y_{1}}{s_{1}}\right)y_{2}=k_{2}y_{2}\left(1-\frac{y_{2}}{s_{2}}+\gamma_{2}\frac{y_{1}}{s_{1}}\right) \end{array}\right.$$ (3)

where *γ*_1_ represents the impact coefficient of URI development scale on urban-rural transport network scale, and *γ*_2_ represents the impact coefficient of urban-rural transport network scale on URI development scale.

By solving the Eqs (1) an (3), the growth trend of urban-rural transport network scale can be found, meanwhile, the stability and elasticity analysis may help determine the optimal transport network scale.

## Results and discussion

**Fig 1:**
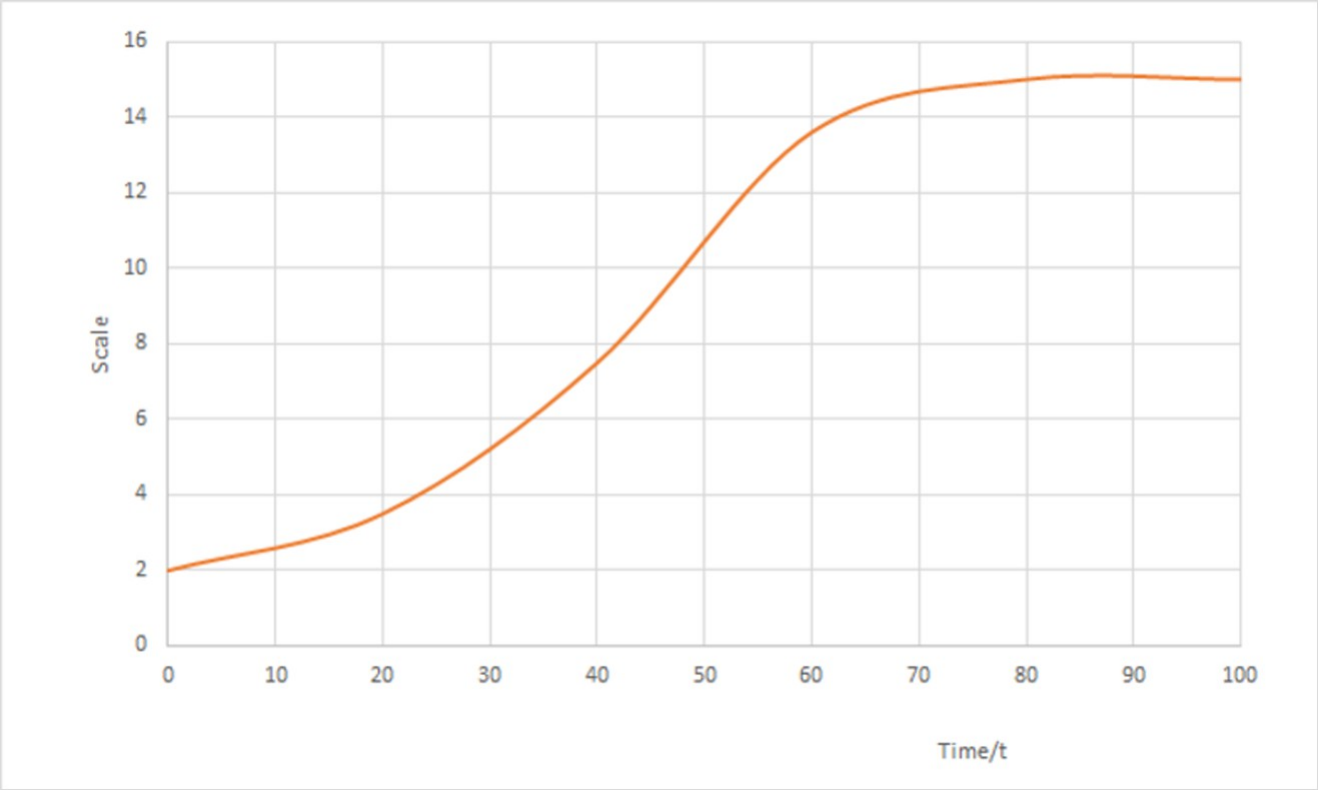
Evolution shape of urban-rural transport network.

According to Eq (1), the evolution curve of urban-rural transport network under certain growth limitation condition can be obtained as Fig 1, in which x-axle stands for time and y-axle is for scale.

Shown from Fig 1, generally speaking, the individual evolution trend of URI scale and urban-rural transport network scale is more like S-shaped curve, i.e. after the slow developing period, it will be increased to the maximum when the certain proportion of maximal threshold is reached, and then the trend tends to decrease due to various influencing factors. For instance, when the transport network scale approaches to the limit *s*, the model parameters may be changed by accompanying social development, adjustment of general urban-rural planning scale and other factors, which may cause the model to start a new developing period. Therefore, URI scale and transport network variation can be interactive with each other.

Macroscopically, there exists periodical developing features for both urban-rural transport network and URI scale before they reach dynamic equilibrium. In detail, during each developing stage of urban-rural transport network and URI scale, the impact factor between them varies gradually with the development, but when both approach to the maximal values, the impact factor becomes weak while a dynamic equilibrium relation is always accompanied. After the maximal values are reached, both may begin with next developing stage influenced by technology, economy and environment factors, and may reveal identical developing features. To sum up, the development between both is a continuous interaction and developing process, and realizes the process from low level to high level gradually.

Next, stability analysis is required when the interaction process reaches the stable state, i.e. when *t* → ∞, the stability can be obtained from the variation trend of *y(t)*. Since the right sides of Eq (3) don’t include time *t*, let them equal to zero, and the solutions can be considered to perform conduct stability analysis.

$$\left\{\begin{array}{c} k_{1}y_{1}\left(1-\frac{y_{1}}{s_{1}}+\gamma_{1}\frac{y_{2}}{s_{2}}\right)=0\\ k_{2}y_{2}\left(1-\frac{y_{2}}{s_{2}}+\gamma_{2}\frac{y_{1}}{s_{1}}\right)=0 \end{array}\right.$$ (4)

By solving the Eq (4), four equilibrium points can be gotten, A (0, 0), B (*s*_1_, 0), C (0,*s*_2_), and D ($s_{1}\frac{1-\gamma_{1}}{1-\gamma_{1}\gamma_{2}},s_{2}\frac{1-\gamma_{2}}{1-\gamma_{1}\gamma_{2}}$), respectively. If the four points are put in the coordination, the equilibrium point is meaningful only in the first quadrant. Apparently, points A, B and C are not meaningful while point D is only deserving when *γ*_1_*γ*_2_ < 1.

There are three conditions of *γ*_1_*γ*_2_ < 1 to determine the stability:

1. Case 1, *γ*_1_ < 1, *γ*_2_ < 1 represents that URI scale and urban-rural transport network scale are boosted for each other, and both are developed collaboratedly. In this case, both scales are in harmonious states and can promote each other;
2. Case 2, *γ*_1_ > 1, *γ*_2_ < 1 represents that URI scale gives more impetus to urban-rural transport network scale. In this case, URI development brings the expansion of transport network, whereas urban-rural transport network is adapted to the sprawling of URI scale passively;
3. Case 3, *γ*_1_ < 1, *γ*_2_ > 1 represents that urban-rural transport network development gives more impetus to URI scale, and stimulates the URI effectively. In this case, transport network construction leads and promotes the URI development, and transport network is surpassed properly.

By selecting density of urban-rural transport network and approximate radius of URI scale as indexes, simulation is conducted with MATLAB software. It is assumed that the initial density of urban-rural transport network is 2km/km^2^ with upper limit 5.3km/km^2^ (according to the lower limit of Standard for urban comprehensive transport system planning) [15], the radius of URI scale is set as 10km and the radius of URI development is set as 20km. Table 1 gives the parameter selection values of equilibrium points.

**Table 1:** Parameter selection value of equilibrium points.

No.	Parameter setting	*γ*_1_	*γ*_2_	*γ*_1_ *γ*_2_
**Case1**	*γ*_1_ < 1, *γ*_2_ < 1	0.4	0.5	0.20
**Case2**	*γ*_1_ > 1, *γ*_2_ < 1	1.5	0.5	0.75
**Case3**	*γ*_1_ < 1, *γ*_2_ > 1	0.4	1.5	0.60

**Fig 2:**
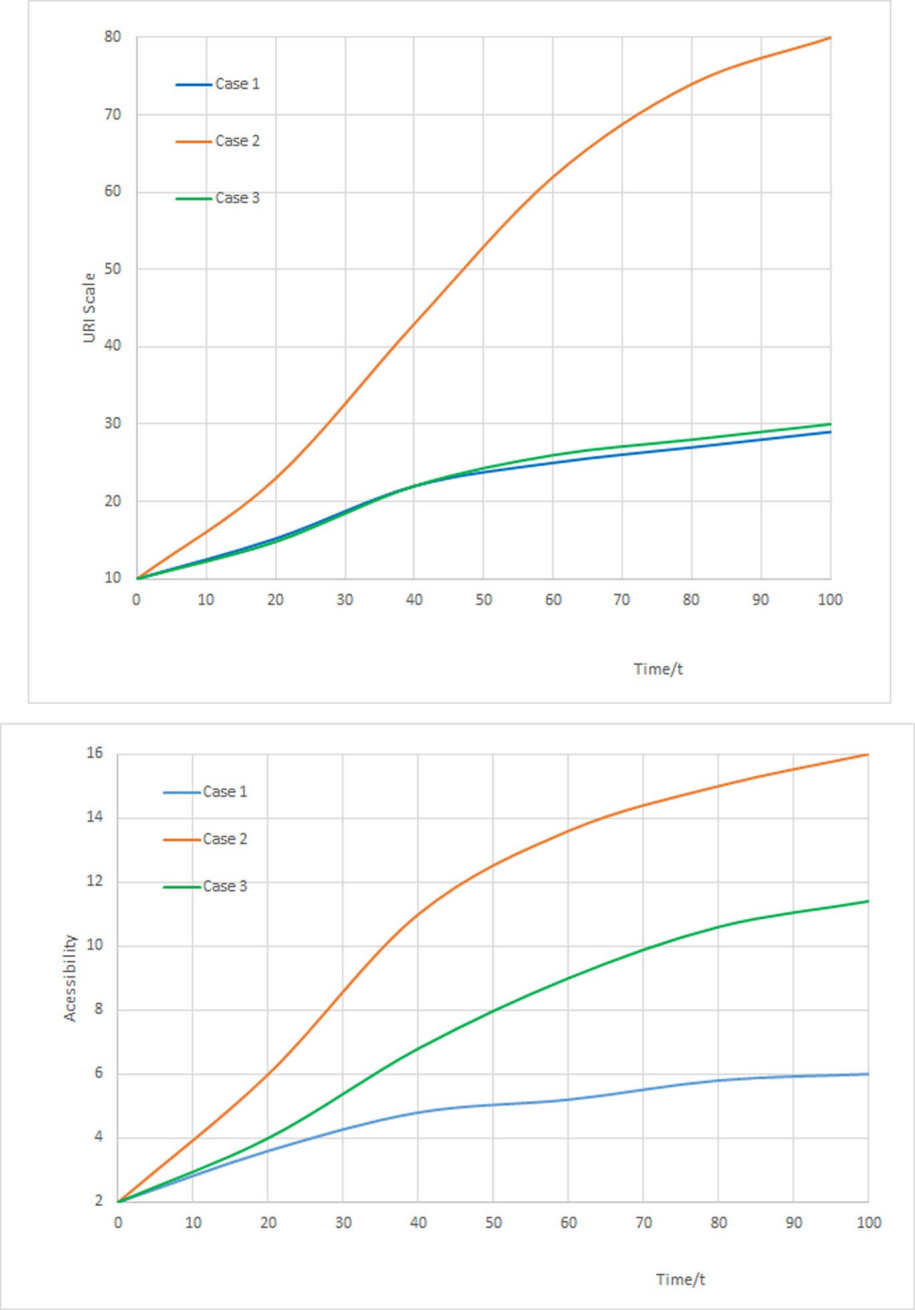
Simulation results at equilibrium points.

In terms of the parameter values and the model, the evolution simulation results are displayed as follows:

Revealed from the Fig 2, in case 1, URI scale matches with urban-rural transport network accessibility normally, and the convergence trend is a little larger than the preset goals of URI scale and transport network scale, which caters to the reasonable urban-rural development planning and transport network development. This case can be considered as ideal interactive and stable development principle.

In case 2, URI space development dominates transport network and its rapid expansion, and surpasses the convergence goals. In this case, the sensitivity of URI scale on accessibility is strong, which boosts the transport network scale to expand passively.

In case 3, urban-rural transport network accessibility dominates the URI space, indicating that transportation stimulates the URI development. When transport network scale is increased rapidly, URI developing speed may rise significantly, and the growth goals may exceed convergence speed. In this case, transport pattern, e.g. transit-oriented development (TOD), may be the representative.

Shown from three cases above, there exists the evolution principles for both URI scale and transport network scale, and the developing speeds are varied constantly. The elasticity variation curve can be employed to reflect the impact of transport network scale on URI scale.

For two adjacent evolution periods, URI scale and transport network scale can be expressed as follows:

$$\text{E}\left(\text{t}\right)=\frac{\left[y_{1}\left(t\right)-y_{1}\left(t-1\right)\right]/y_{1}\left(t-1\right)}{\left[y_{2}\left(t\right)-y_{2}\left(t-1\right)\right]/y_{2}\left(t-1\right)}$$ (5)

where E(t) is the elasticity of transport network scale within evolution period *t*.

**Fig 3:**
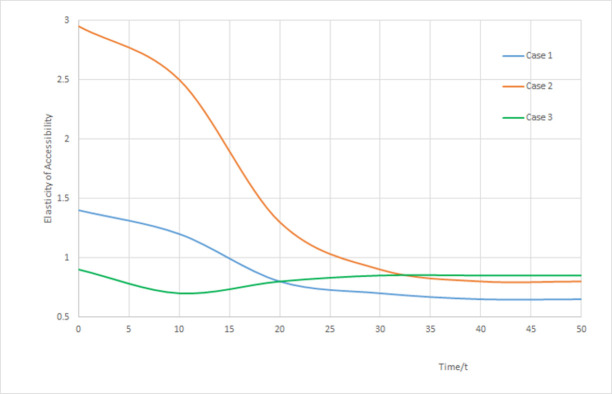
Elasticity results of accessibility.

With three cases above, the elasticities of transport network scale at equilibrium points can be performed with MATLAB simulation as shown in Fig 3.

Shown from curves of Fig 3, elasticities of transport network scale converges between 0.5 and 1.0 and approaches to 1.0, which implies that in the end of developing stage, the impact of URI scale from transport network would be reduced, and both increasing trend tends to be identical, while other factors may have some influence.

In case 1, the initial state of transport network elasticity is larger than 1.0, and then converges gradually. This reflects that when transport restricts regional development, increasing transport network scale will improve the URI scale speed, but the influence will be decreased step by step. This case is suitable for simulating the normal development of URI scale and urban-rural transport network.

In case 2, the initial value of elasticity is large, and then it is decreased to converge, which reflects that enlargement of URI scale promotes the transport network expanded. In case 3, the elasticity values are smaller than 1.0, and then converged gradually, implying that transport network is developed in advance, and then boosts URI development. This case is suitable for the pattern of transport network dominating URI scale.

By summarizing the analysis above, it can be found out that there exists an interactive relationship between URI scale and urban-rural transport network, and stability analysis verifies that there exists certain dynamic equilibrium. Known from the assumptions of the models, URI development has a significant impact on transport network, mainly featuring growth scales *s*_1_, *s*_2_ and interaction coefficients *γ*_1_*γ*_2_. In the simulation, hypothetical *s*_1_, *s*_2_ and *γ*_1_*γ*_2_ are adopted, and different *s*_1_, *s*_2_ values may reflect different developing stages and patterns.

Empirically, there are some issues to be discussed. First, restrained by a variety of factors, such as economy, social development, politics, etc., URI and transport network have certain limitation, but under certain conditions, the interaction can reach the dynamic equilibrium; Second, when the assumptions or restraints can’t meet the requirements, the development of both sides may not be limited, and the trend may be varied randomly, which may lead to unharmonious phase. Third, revealed from the stability analysis and simulation results, it can be found that the accessibility of transport network converges around 0.85 of stable value, which can be considered as the optimal value of transport network scale configuration. At last, the interaction between URI and urban-rural transport network is effective within certain range, and when high-intensity land use produces high-density travel demand, the interactive relation may be broken, and the evolution theory and models above may not be suitable any more.

## Conclusions

With the progress of urbanization and URI, the interaction between URI and urban-rural transport network is becoming stronger, thus it is necessary to construct an interactive model between URI and transport network. By assuming certain functions and principles, evolution model was proposed to explain the relationship between URI space development scale and urban-rural transport network scale, and then by considering the interaction between URI and transport network, dynamic evolution model was presented. Stability analysis was performed to verify the interaction relations with three patterns and elasticity simulation provided the optimal value of transport network scale configuration.

Two main findings can be obtained from the results of the work. First, dynamic evolution model not only offers the relationship between URI space development and urban-rural transport network, but also emphasizes the interaction between them. This is to our knowledge the first attempt to explore their relationship and expands the range of URI analysis. Second, elasticity simulation presents the optimal value of transport network scale configuration, which can be considered as an objective to determine urban-rural transport network scale.

Some drawbacks may need to be strengthened in the further study. As stated above, there are a variety of factors influencing URI, and more parameters should be added in the model so that the relations may be reflected completely. Another issue is that the results of the work are founded on the simulation, and it is worthy of employing actual data sources to ascertain the findings and transferability in the future. Future study may consider the URI spatially and temporally, so that spatial and temporal issues can be addressed clearly.

## Supporting information

S1 Data (XLSX)
